# Edge treatment for spurious mode suppression in thin-film lithium niobate resonators

**DOI:** 10.1038/s41598-024-71036-8

**Published:** 2024-09-09

**Authors:** Arjun Aryal, Sidhant Tiwari, Darren W. Branch, Aleem Siddiqui, Tito Busani

**Affiliations:** 1 Center for High Technology Materials (CHTM), University of New Mexico (UNM), MSC01 04-2710, 1313 Goddard SE, Albuquerque, NM 87106-4343 USA; 2 Sandia National Laboratories (SNL), 1515 Eubank SE, Albuquerque, NM 87123 USA; 3 Optical Sciences and Engineering (OSE), University of New Mexico (UNM), MSC01 04-2710, 1313 Goddard SE, Albuquerque, NM 87106-4343 USA; 4 Electrical and Computer Engineering (ECE), University of New Mexico (UNM), MSC01 11001, Albuquerque, NM 87131-0001 USA

**Keywords:** RF acoustic devices, Electromechanical coupling, Spurious modes suppression, Edge treatment, Etch process, Lamb wave devices, Nanoscience and technology, Engineering, Electrical and electronic engineering, Physics, Acoustics

## Abstract

Thin-film lithium niobate is an attractive material for RF acoustic devices because of its high electromechanical coupling. However, due to the large coupling and the high anisotropy, thin-film lithium niobate resonators are prone to accidental resonances called spurious modes. These modes compromise the frequency response of the resonators, limiting their use in filter and oscillator applications. In this work, we present a novel method of spurious mode suppression through a special edge treatment etch process. Two thin-film lithium niobate resonators were fabricated, one with smooth sidewalls and one with the edge treatment. It was found that the edge-treated resonators show a weaker spurious mode response. This is potentially a new way to mitigate spurious resonances, a major issue in lithium niobate Lamb wave devices.

## Introduction

**Fig. 1:**
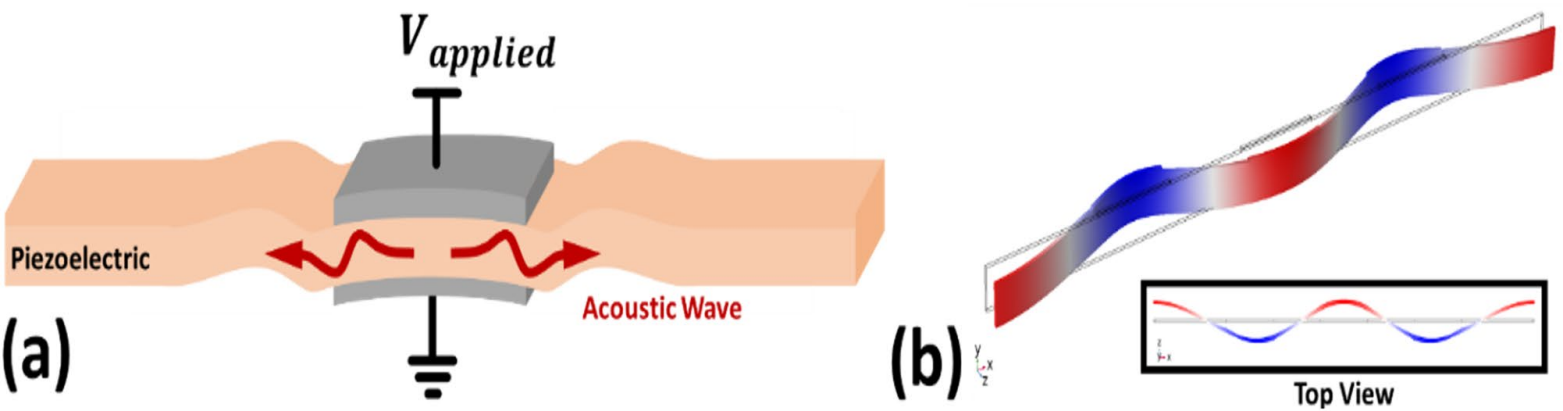
(**a**) An oscillating voltage is applied to a piezoelectric slab, generating acoustic waves that propagate away from the electrodes. (**b**) When the frequency of the voltage matches that of the slab mechanical resonance mode, the generated acoustic waves constructively interfere with previously generated acoustic waves that were reflected off the boundaries and mechanical resonance is excited.

Piezoelectric resonators are key components in modern day electronics. Originally developed as high-stability frequency references for AM radio stations in the 1920s^1^, piezoelectric resonators now have applications as sensors^2–4^, filters^5–7^, clocks^8^, and ultrasonic transducers^9^. By leveraging the piezoelectric effect, these devices convert applied voltages to strains (Fig. 1). In the case of an alternating voltage, acoustic waves are generated. These acoustic waves reflect off the boundaries of the device, causing resonance when the reflected waves constructively interfere with the original waves. The specific resonance mode and frequency depends on the geometry of the device. For example, frequencies of bulk acoustic wave (BAW) resonators^10^ typically are set by their thickness, contour and Lamé mode^11,12^ resonators by their width, and Lamb and surface acoustic wave (SAW)^13^ resonators by their electrode pitch.

**Fig. 2:**
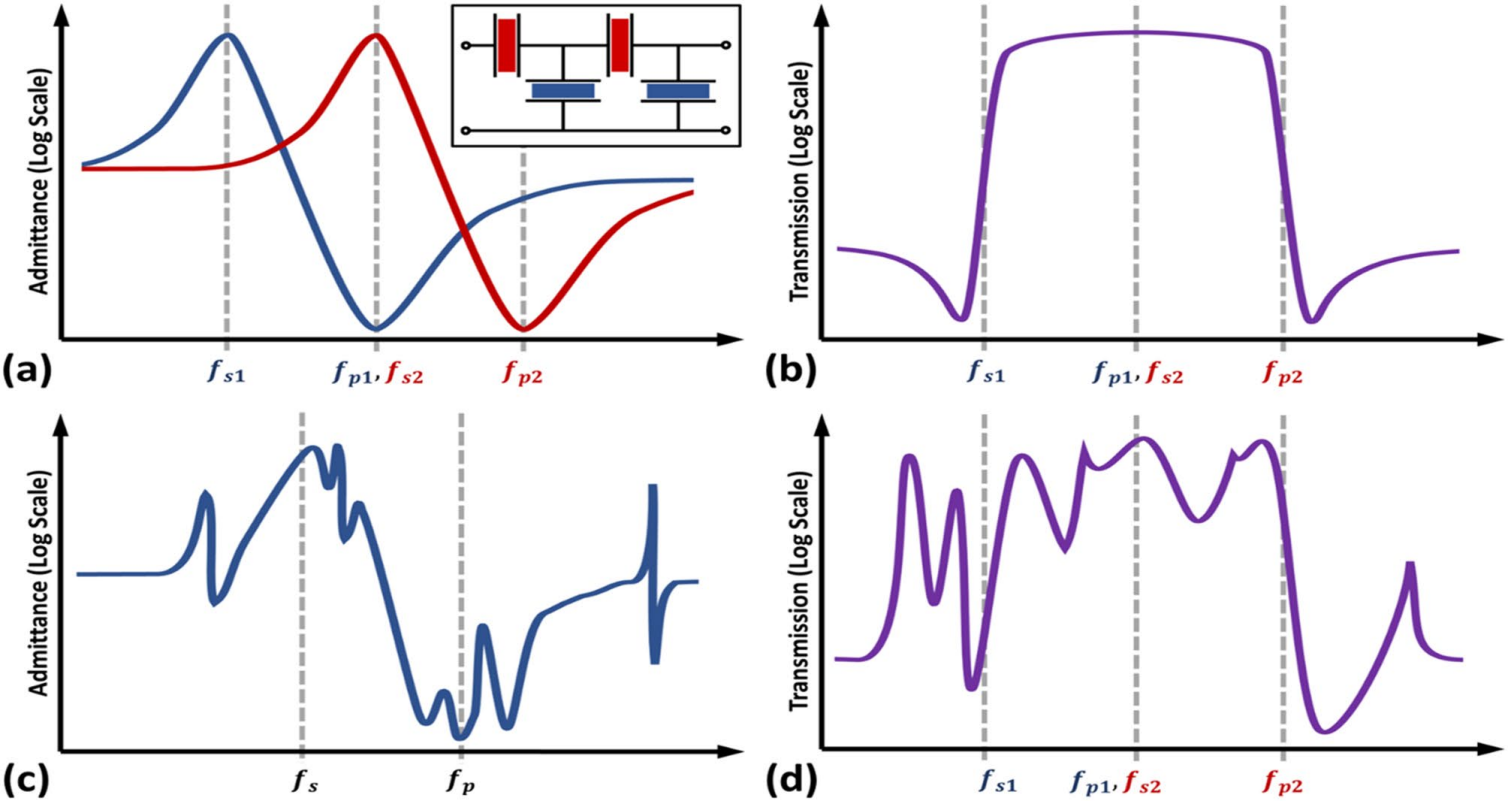
(**a**) Admittances of series (red) and shunt (blue) elements of a piezoelectric filter as a function of frequency. The electromechanical coupling factor determines the separation of the high admittance point (f_s_) and the low admittance point (f_p_), with a higher coupling factor increasing the separation. A higher Q factor makes the transition between f_s_ and f_p_ more abrupt. (**b**) Ideal filter response. A higher electromechanical coupling increases the bandwidth of the filter, and a higher Q factor increases the sharpness of the filter passband. (**c**) Influence of spurious modes on piezoelectric resonator admittance, where additional modes are superimposed over the expected resonator response. (**d**) Influence of spurious modes on filter response.

Acoustic wave-based resonators are significantly more compact than their electromagnetic equivalents at the same frequency. This size advantage has made them useful in applications like mobile phone filters^10^ and it has motivated the development of thin-film piezoelectric devices, where the coupling between voltage and strain is notably strong^14,15^. High electromechanical coupling factor and high-quality factor (Q factor) are necessary for optimal piezoelectric filter performance (Fig. 2a,b). Several design techniques have been proposed for coupling factor^16,17^ and Q factor enhancement^18,19^ in piezoelectric resonators, but the best these techniques can do is approach the material limits. For this reason, much of the recent work on 5G compatible piezoelectric resonators has been focused on ion-sliced thin-film single-crystal lithium niobate (Table 1). Due to being single crystalline, the Q factors of lithium niobate exceed that of the polycrystalline piezoelectric thin films, with Q factors of up to 30,000 demonstrated in bulk crystals^20^. Lithium niobate is among the highest coupling factors of all piezoelectric resonators, with resonators having demonstrated coupling coefficients in excess of 40%^21^.

**Table 1:** State-of-the-art $k_{t}^{2}$ and Q performance for thin-film lithium niobate MEMS or acoustic resonators.

Material	Thickness of LN film (nm)	Mode	f (GHz)	${\text{k}}^{2}$(%)	Q	f*Q (*e12)	References
Y-cut	1200	A1	1.70	6.30	5341	9.079	^39^
Z-cut	400	A1	4.50	24.00	118	0.531	^39^
Z-cut	400	A3	12.90	3.70	224	2.890	^39^
Z-cut	400	A5	21.40	1.50	287	6.141	^39^
Z-cut	400	A7	29.90	0.95	328	9.807	^39^
Y-cut	1200	A1	1.65	14.00	3112	5.135	^25^
X-cut	2000	S0	0.05	27.80	5329	0.266	^40^
128Y-cut	550	A1	3.20	46.40	598	1.913	^38^
128Y-cut	550	A3	9.55	5.66	359	3.428	^21^
128Y-cut	550	A5	15.90	2.26	321	5.202	^21^
128Y-cut	550	A7	22.20	1.14	294	6.526	^21^
Z-cut	400	A1	4.40	15.00	50	0.220	^41^
Z-cut	400	A3	12.90	1.90	360	4.644	^41^
Z-cut	400	A5	21.60	1.00	520	11.232	^41^
Z-cut	400	A7	30.10	0.72	670	20.167	^41^
Z-cut	400	A9	38.70	0.63	570	22.059	^41^
Z-cut	400	A3	13.00	3.80	372	4.836	^41^
Z-cut	400	A5	21.60	1.20	566	12.220	^41^
Z-cut	400	A7	30.20	6.30	715	21.590	^42^
Z-cut	400	A9	38.80	14.00	539	20.910	^42^
Z-cut	400	A11	47.40	24.00	474	22.460	^42^
Z-cut	400	A13	55.00	3.70	340	18.700	^42^
Z-cut	400	A1	5.00	28.00	171.7	0.860	^43^
36YX-cut	300	SH0	1.10	28.00	600	0.660	^44^
ZY-cut	300	A1	5.89	14.00	300	1.767	^45^
Z-cut	300	A1	6.00	26.00	1123	6.738	^46^
Our work							
Y-cut	2000	SH0	0.103	10.9	37.1	0.004	Smooth sidewall
				10.8	82.6	0.009	Rough sidewall
Y-cut	2000	SH0	0.206	15.6	1468.5	0.302	Smooth sidewall
				15.0	1239.9	0.277	Rough sidewall
Y-cut	2000	SH0	0.319	1.1	2279.2	0.727	Smooth sidewall
				1.0	540.3	0.187	Rough sidewall
Y-cut	2000	A1	0.779	3.7	1557.5	1.213	Smooth sidewall
				7.2	123.5	0.094	Rough sidewall
Y-cut	2000	A1	0.837	3.0	3486.0	2.917	Smooth sidewall
				3.9	660.5	0.541	Rough sidewall
Y-cut	2000	A1	0.864	3.5	1394.3	1.205	Smooth sidewall
				2.2	1067.6	0.912	Rough sidewall

One of the major challenges in the adoption of thin-film lithium niobate piezoelectric resonators is that of spurious resonant modes. Due to the high anisotropy, the high coupling, and the low losses of lithium niobate, resonances outside of the target mode are easy to excite. When these resonances are near the target mode, they introduce numerous spikes into the filter transfer function (Fig. 2c,d), which either complicate or degrade filter performance. Several methods to mitigate spurious mode in thin-film lithium niobate have been investigated, such as anchor shaping^22^, acoustic reflectors^23^, electrode optimization and device arraying^24–27^.Thus, designing robust filters for ultrahigh frequency (UHF) and very high frequency (VHF) bands continues to remain challenging, highlighting the need for alternative design approaches to overcome the limitations present in the RF spectrum, including UHF, VHF, and 5G^28–32^. By focusing on novel fabrication processes, it is possible to achieve resonator designs that operate effectively in the UHF/VHF range, and which can be scaled up to 18.5 GHz and beyond.

In this work, we present a novel method to mitigate spurious modes through fabrication process design rather than optimizing the device geometry. Thin-film lithium niobate resonators are fabricated using a previously developed high-aspect-ratio lithium niobate dry etching process^33^. The free edges of the resonators are subsequently treated with a targeted etch process to enhance the wave scattering^34^. We believe the additional wave scattering disrupts the spurious modes, preventing them from forming strong resonances and resulting in a smoother resonator frequency response. This is a particularly counter-intuitive result as smooth, high-aspect-ratio sidewalls are typically expected to give the best device performance. We evaluate the effectiveness of this by suppressing spurious or reducing the intensity of modes up to 18.5 GHz, anticipating significant potential as we continue to refine and scale this methodology, for integration into the 5G frequency range.

### Device fabrication and edge treatment process

**Fig. 3:**
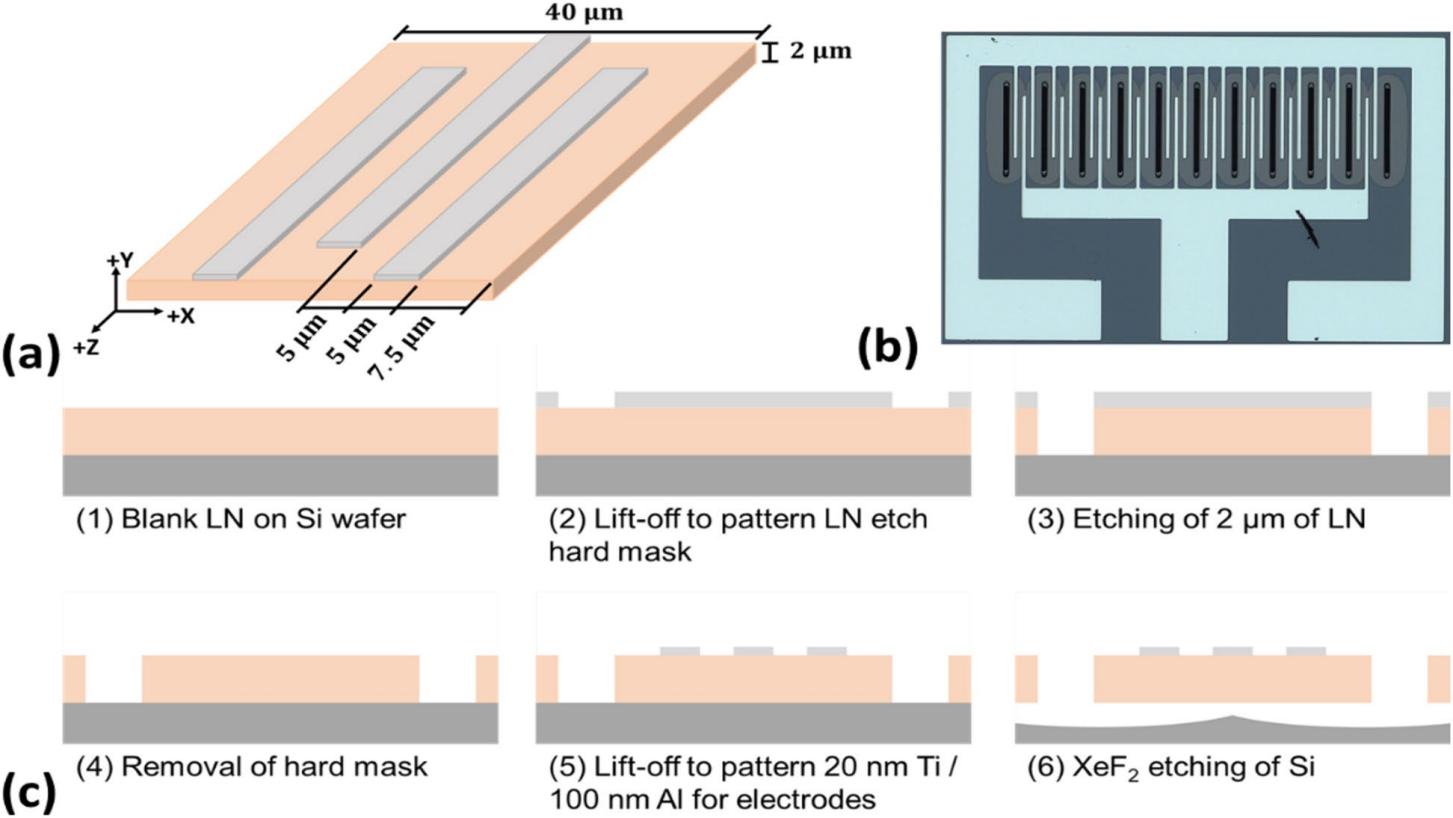
(**a**) Diagram of lithium niobate (LN) resonator design. (**b**) Microscope image of a fabricated device. (c) Device fabrication process flow.

A nominal design for the resonator was established as a baseline, upon which we implemented our innovative fabrication process to enhance performance characteristics. This foundational design served as a control to rigorously evaluate the impact of our novel approaches, ensuring that any observed improvements could be directly attributed to the specific fabrication modifications applied. A schematic of the designed resonator is shown in Fig. 3a. The device is a center-symmetric three finger Lamb wave resonator on Y-cut lithium niobate, with the electrode fingers perpendicular to the lithium niobate X-axis, and the finger widths and the finger gaps are both 1/8th of the plate width. Several resonator elements are cascaded in parallel to increase the total device admittance. An image of a fabricated device is shown in Fig. 3b. Two sets of Lamb wave resonator devices were fabricated for comparison: one with high quality “smooth sidewalls”, and another with a “rough sidewall” that was exposed to an etch treatment.

**Fig. 4:**
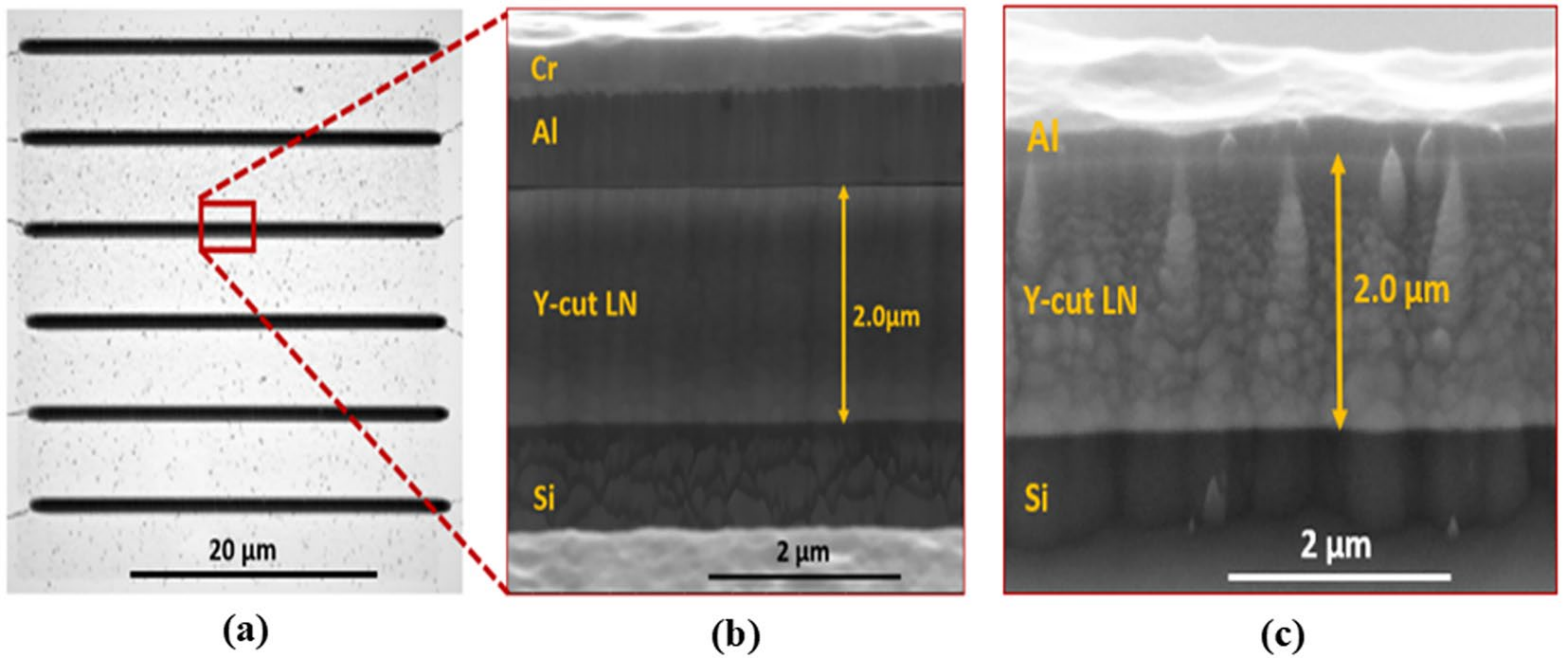
(**a**) Top view of a typical etched structure using CHF3/Ar plasma. (**b**) Side view of the etched structure using Ti/Al/Cr as hard mask and (**c**) using Ti/Al mask as hard mask.

Figure 3c shows the fabrication process for the device, full details of which can be found in the supplementary documentation (Supplementary Fig. S1). A 2 μm Y-cut lithium niobate thin-film on Si substrate was first immersed in piranha solution (H_2_SO_4_: H_2_0_2_, 4:1) for 10 min, then immediately exposed to H_2_ plasma, as described in previous work^34^. The H_2_ plasma was used to improve the adhesion between the LN film surface and the deposited metal hard mask used for the etching process. A negative photoresist (AZ nLOF 2035) mask was prepared (Supplementary Fig. S2) for lift-off patterning of the metal hard mask. Patterned samples were cleaned by immersion into HCl: H_2_O, 1:3 ratio, before metal deposition. A Ti/Al/Cr hard mask was then deposited, using an e-beam evaporator at a pressure of 1.0 × 10^−6^ Torr. After lift-off (Supplementary Fig. S3), the samples were then etched using a Plasma-Therm Inductively Coupled Plasma (ICP) using CHF_3_/Ar as precursor gases. A top view of the etched structures of the Lamb wave resonators is shown in Fig. 4a. The resulting “smooth side wall” is shown in Fig. 4b. Using a Ti/Al hard mask rather than Ti/Al/Cr, and applying the etching recipe that was used previously, we were able to produce rough sidewall surfaces as shown in Fig. 4c. Al reacts with the fluorine process much faster than the Cr resulting in a poor-quality sidewall etch. After the dry etching, the hard mask was removed (Supplementary Fig. S4) using standard Cr etchant and 6.25% HF diluted in H_2_O respectively for the Cr and the Ti/Al. Next, 20 nm/100 nm of Ti/Al was deposited as the device electrode metal, using an electron bean evaporator at ~ 10^−6^ Torr partial pressure. The devices were then released using XeF_2_ dry vapor to etch partially the Si underneath the resonator body. The comparison of Fig. 4b,c illustrates that we achieved the desired roughened edges.

### Characterization results

**Fig. 5:**
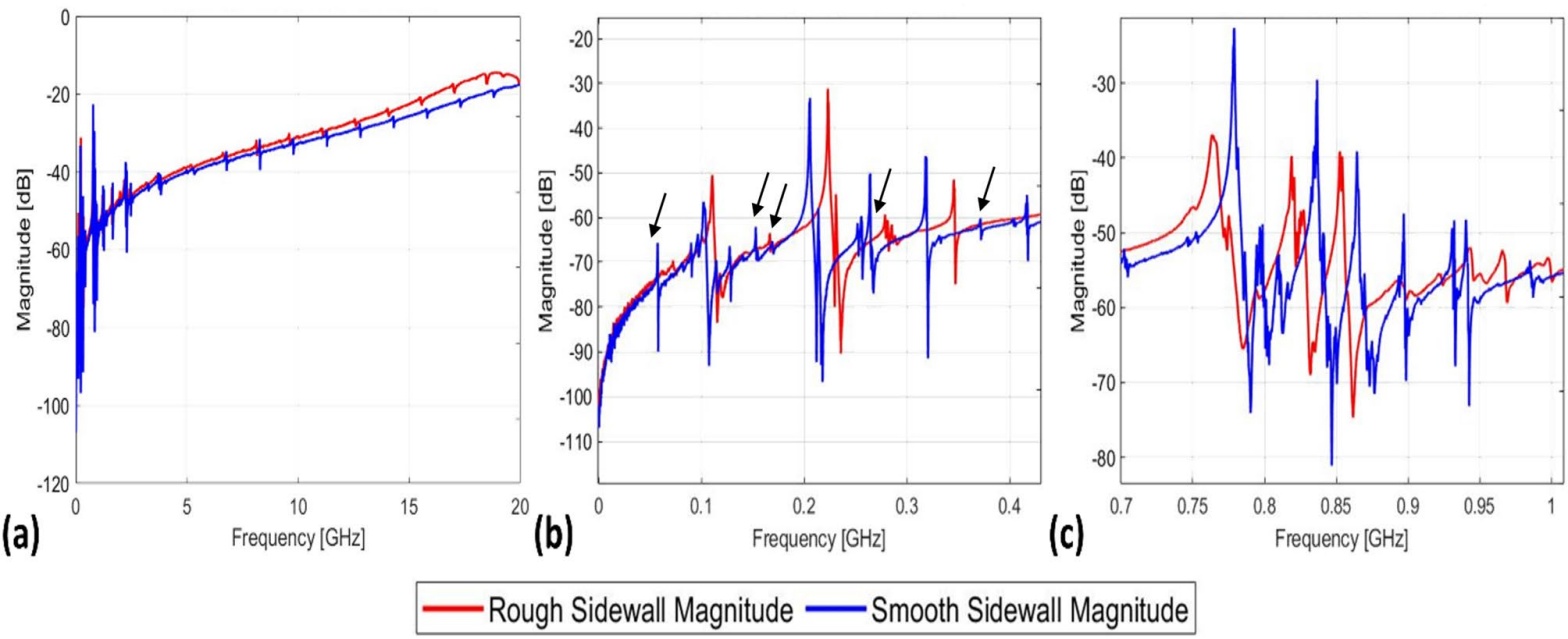
Measured admittance magnitudes for fabricated thin-film lithium niobate resonators. (**a**) Full frequency sweep of measured devices. (**b**) Lower frequency modes from 300 kHz to 400 MHz. (**c**) Lower frequency modes from 700 MHz to 1 GHz.

The fabricated devices were tested and characterized at room temperature using a Keysight P9374A network analyzer after performing a Short-Open-Load-Through (SOLT) calibration of the Ground-Signal-Ground (GSG) probes (GGB Industries, Model 40A). Measurements were acquired over the full range of the network analyzer, 300 kHz to 20 GHz. The result shown in Fig. 5a compares the response of devices with smooth sidewalls and devices with side walls roughened by the edge treatment. We were able to excite multiple resonances corresponding to acoustic modes supported by the device, and the highest resonant frequency we could measure with our current set up was found to be 18.5 GHz. Zoomed in views of the responses of each mode are shown in the Supplementary Documentation (Supplementary Fig. S5). We observed that the resonant frequencies of all the target modes are all slightly shifted between the two devices. This can be either due to die-to-die process variations or the fact that the edge treatment not only roughens the edge but removes the material, perturbing the resonator geometry.

Supplementary Fig. 5b,c show the resonant modes of interest for this work. The modes in Fig. 5b (103 MHz, 206 MHz, and 319 MHz ) are identified to be shear horizontal (SH0) modes and the modes in Fig. 5c (779 MHz, 837 MHz, and 864 MHz) are identified to be first-order antisymmetric (A1) modes. The smooth sidewall devices, shown in blue, are riddled with several weaker spurious modes throughout the frequency range. The roughened sidewall devices, shown in red, show a smoother frequency response. In the low frequency range (Fig. 5b), several spurious modes, respectively at 50 MHz, 150 MHz, and 365 MHz are completely removed by the edge treatment process, while the peaks at 175 MHz and 250 MHz are greatly suppressed. Those peaks are marked with an arrow in Fig. 5b. The magnitude of the strongest resonance near 200 MHz is barely changed by the edge treatment. As stated earlier in this work, this is a counterintuitive result as edge roughening is typically not seen as a way to improve resonator performance. We hypothesize that the spurious modes in the 100 to 300 MHz have shorter wavelengths than the main 200 MHz mode and are thus much more easily scattered by the small features introduced by the edge roughening.

In contrast, all modes in the 700 MHz to 1 GHz range (Fig. 5c) are degraded due to the edge roughening with only the three strongest modes (approximately 760 MHz, 820 MHz, and 850 MHz) remaining after the edge treatment. This is consistent with the hypothesis that the edge roughening more strongly affects smaller wavelength modes. For example, the scattering loss $\alpha_{s}$ due to Rayleigh scattering^35^ is estimated as:

1 $$\alpha_{s} = \left( {4\pi \sigma / \lambda } \right)^{2}$$

where *σ* is the root mean square (RMS) surface roughness and *λ* is the wavelength of the wave. Thus, since the 750–850 MHz modes have a shorter wavelength than the 200 MHz mode, the edge roughening would be expected to have larger impact on these higher frequency modes.

Multiple resonators were fabricated on the same chip to observe for any statistical variation in the rejection of spurious modes. The frequency responses of all the measured devices are plotted in Supplementary Fig. S6. A total of 20 smooth sidewall devices and 25 rough sidewall devices were measured. There is clearly one rough sidewall device that did not yield, leaving a total of 24 rough side wall devices.

To help qualitatively see trends in the data, Supplementary Fig. S7 plots the resonator responses overlaid on top of each other with transparent traces. When multiple traces overlap, the region darkens. The darker the region, the less statistical variation in that area. It is clear comparing the plots for the smooth and rough sidewalls, that the rough sidewall devices show weaker spurious modes.

Supplementary Fig. S8 quantifies statistical variation in the rough and smooth device measurements. While there is slightly more variation in the rough sidewall devices, the traces marking the upper and lower quartiles in the admittance measurement are very close to the median. This indicates excellent repeatability in the device frequency response and further supports our claim that roughening the edges of our devices weakens the spurious mode response.

The measured peak admittance of the fundamental mode for the device with smooth side was at 200 MHz is consistent with the response simulated in COMSOL Multiphysics which was found to be at 178 MHz (Supplementary Fig. S9).

The Q factor for the main resonance modes is estimated by measuring the 3 dB bandwidth of the series resonances and using Eq. (2), as was done in^26,36^. The electromechanical coupling factors, $k_{t}^{2}$, were calculated using Eq. (3) ^37^. These results are summarized in Table 1 and compared to the state-of-the-art results found in literature for different LN acoustic wave resonators for different crystallographic orientation.

2 $$Q = \frac{{{\Delta }f_{3dB} }}{{f_{s} }}$$

and

3 $$k_{t}^{2} = \frac{{\pi^{2} }}{8}\frac{{f_{p}^{2} - f_{s}^{2} }}{{f_{s}^{2} }}$$

where $f_{s,p}$ = series or parallel resonant frequency of a piezoelectric resonator, and ${\Delta }f_{3dB}$ = the bandwidth between the points where the admittance amplitude has decreased by 3 dB relative to the series resonance admittance peak. It should be noted that the presence of spurious modes distorts the resonance mode’s admittance profile, making accurate estimation of Q and $k_{t}^{2}$ difficult^38^.

In Table 1 we summarized the state-of-the-art $k_{t}^{2}$ and Q and for several LN cuts and compared them to the devices fabricated by our fabrication process for both rough and smooth side walls. As we have not optimized the device geometry, we did not expect improvement beyond the literature values. The values $k_{t}^{2}$ were found to not differ significantly between the rough and smooth sidewall devices. In contrast, the Q factor for several of the modes is found to have been severely degraded by the edge treatment. This is expected, as we expect the roughened sidewalls to scatter acoustic waves. However, our main resonant mode at 206 MHz was found to not be significantly affected, maintaining a high Q factor. Thus, we have demonstrated that the edge treatment process can indeed reduce spurious modes while still producing a quality resonator.

## Conclusion

In this work we present a study on how an edge treatment process influences the spurious mode response of thin-film lithium niobate resonators. Two thin-film lithium niobate resonators are fabricated, one with “smooth sidewalls” and one with “roughened sidewalls”, and their frequency response is characterized. It was found that introducing edge roughness reduced the spurious modes with minimal influence on the main mode. The hypothesis is that the spurious modes are relatively short in wavelength and are more easily scattered by the rough edges, thus preventing the modes from forming a strong resonance. More test structures with controlled roughness need to be fabricated to validate this idea over a wide range of resonances and device architectures. However, this research potentially shows a new method for mitigating spurious mode in Lamb wave resonators, a major challenge that must be resolved for the practical realization of thin-film lithium niobate RF devices for 5G applications.

## Supplementary Information

Supplementary Information.

## Data Availability

Data is provided within the manuscript or supplementary information files.
