# Supplementary material for: Edge treatment for spurious mode suppression in thin-film lithium niobate resonators
Source: Sci Rep. 2024 Sep 9;14:21070. doi: 10.1038/s41598-024-71036-8 (PMC11387820; doi:10.1038/s41598-024-71036-8)
Supplement: Supplementary file 1 — Supplementary Information. [file 41598_2024_71036_MOESM1_ESM.pdf]

Supplementary Information

Edge Treatment for Spurious Mode Suppression in Thin-Film Lithium Niobate Resonators

Arjun Aryal [1,3,†], Sidhant Tiwari [2,†], Darren W. Branch [2], Aleem Siddiqui *[2], Tito Busani * [1,3,4]

[1] Center for High Technology Materials (CHTM), University of New Mexico (UNM), MSC01 04-2710, 1313 Goddard SE, Albuquerque, NM 87106-4343, United States of America.
[2] Sandia National Laboratories (SNL), 1515 Eubank SE, Albuquerque, NM, 87123 United States of America.
[3] Optical Sciences and Engineering (OSE), University of New Mexico (UNM), MSC01 04-2710, 1313 Goddard SE, Albuquerque, NM 87106-4343, United States of America.
[4] Electrical and Computer Engineering (ECE), University of New Mexico (UNM), MSC01 11001, Albuquerque, NM 87131-0001, United States of America.

*Corresponding author: asiddiq@sandia.gov, busanit@unm.edu

† These authors contributed equally to this work.

Experiments and Results:

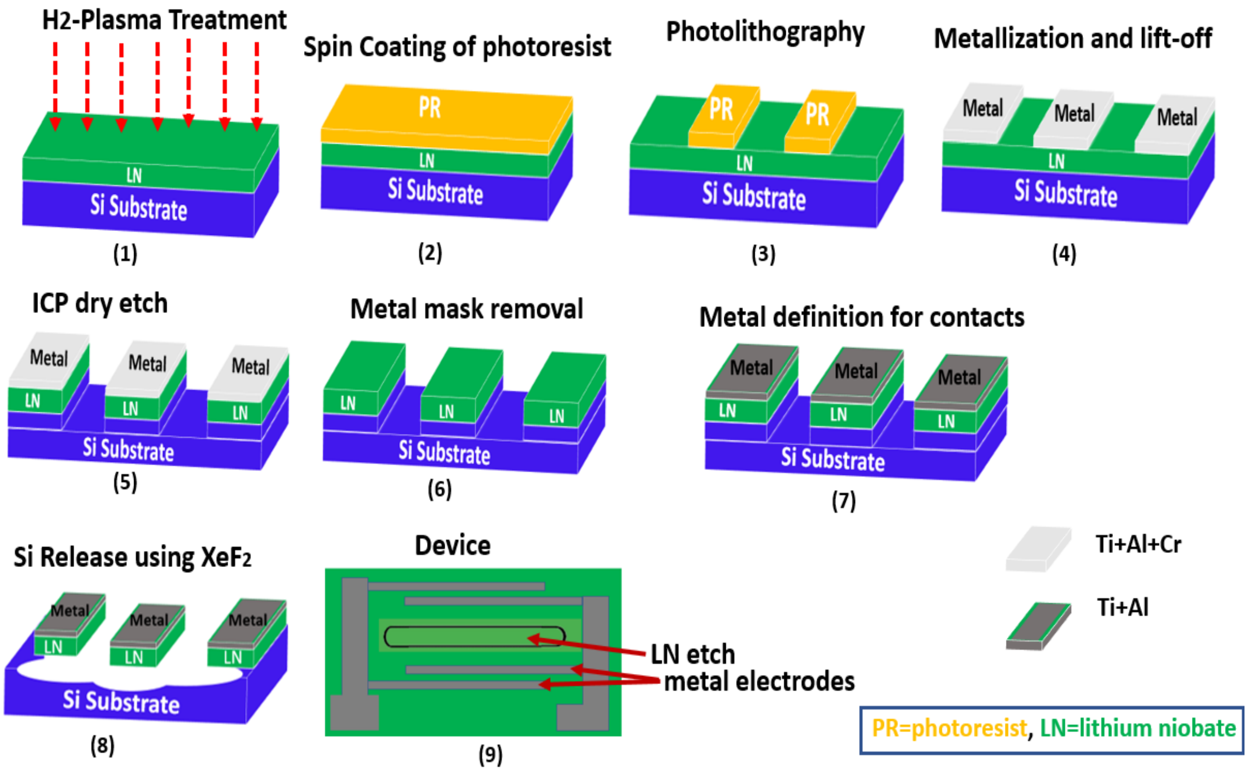

Supplementary Figure S1. Flow chart for the device fabrication process for Y cut LN thin film on Si.

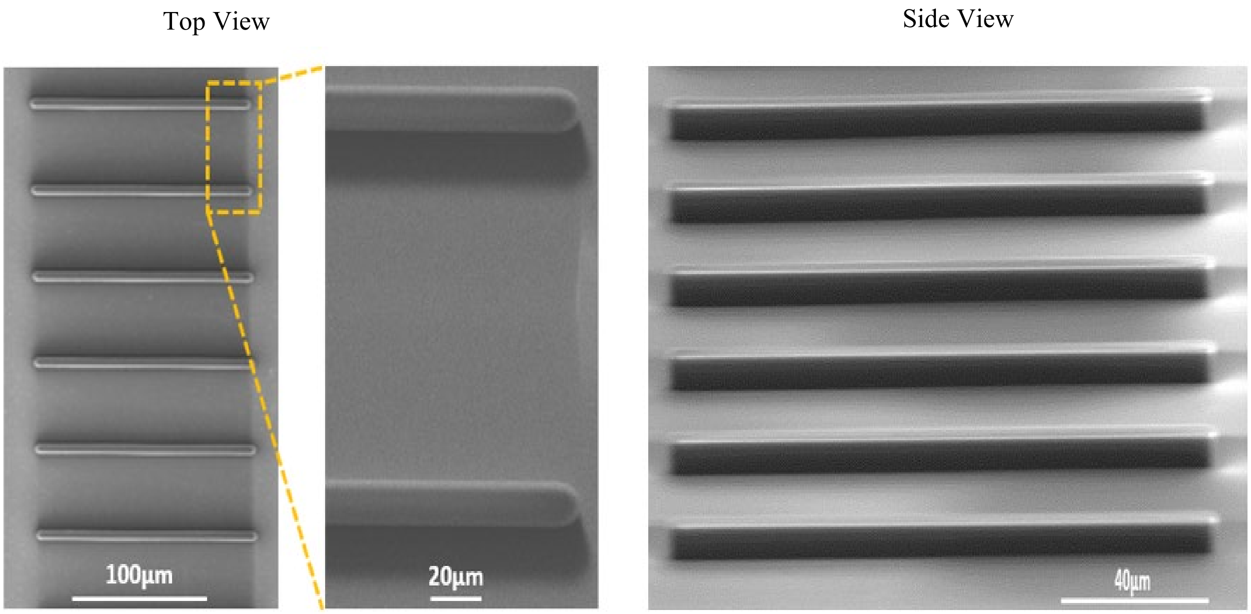

Supplementary Figure S2. SEM image of patterned photoresist after contact lithography.

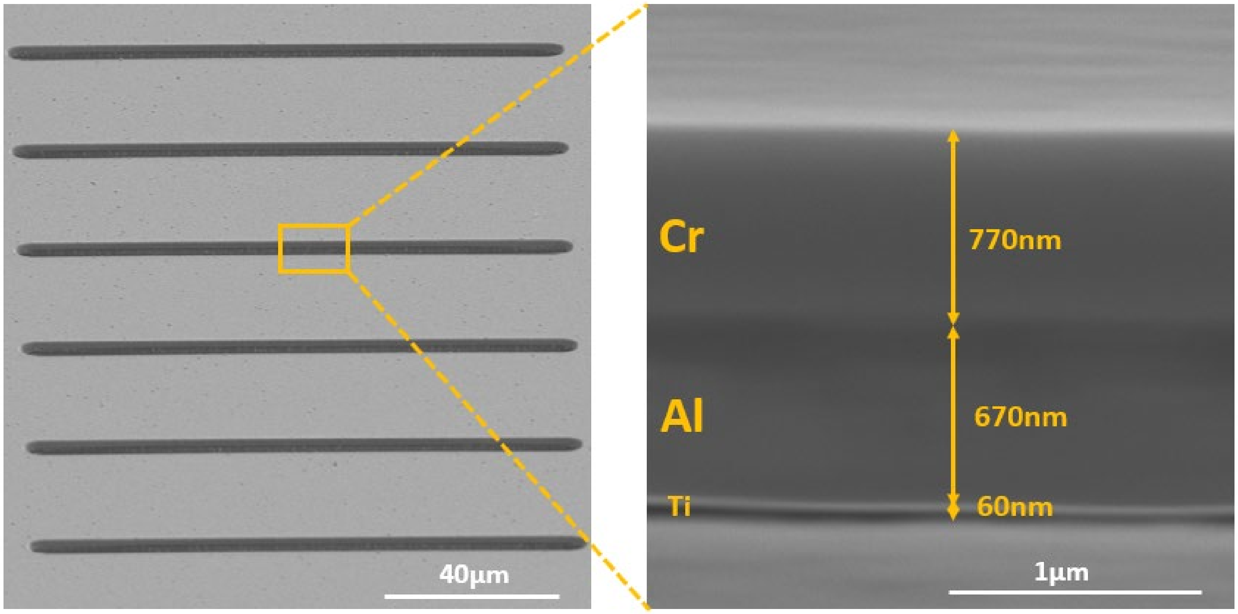

Supplementary Figure S3. Stacks of metals mask defined for dry etch process, LN on Si samples.

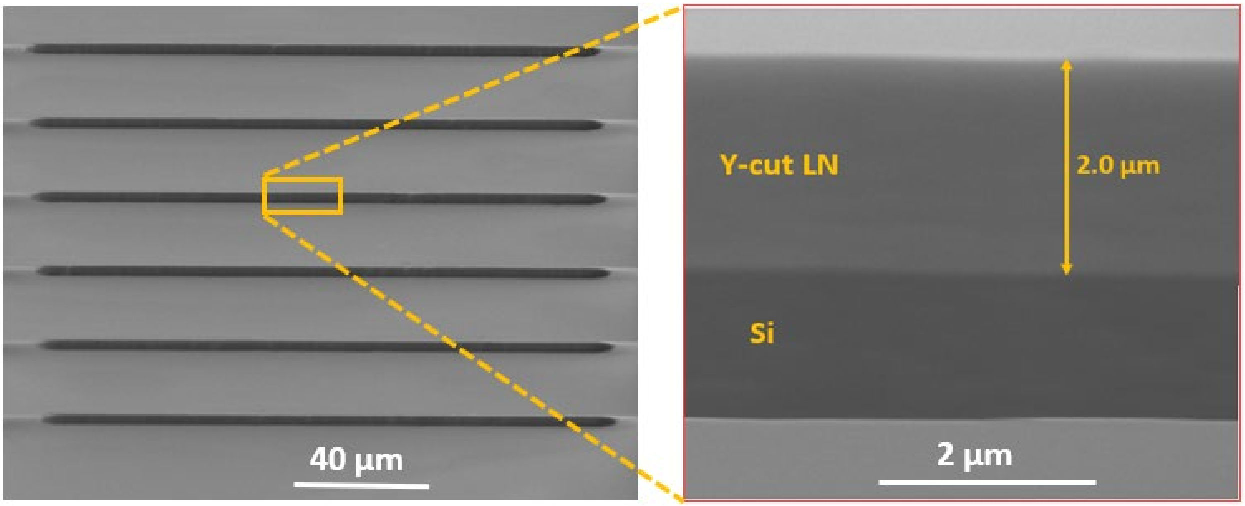

Supplementary Figure S4. Metal masks removal after dry etch process using chrome etchant CEP 200 and diluted HF solution.

Realization of resonating device

The 2nd step of lithography was applied with the same negative photoresist (AZnLOF 2035) to register etch pits between the electrodes. Ti and Al metals stack was deposited to define the electrodes. Ti was utilized as an adhesive layer for Al metal contact. Al metal works as electrodes for the devices. XeF_2 dry vapor was used to etch Si, underneath resonating body for the release process. The XeF_2 vapor doesn't attack Al electrodes as it is extremely high selectivity material for XeF_2 [1].

Measured admittance of the resonators at room temperature

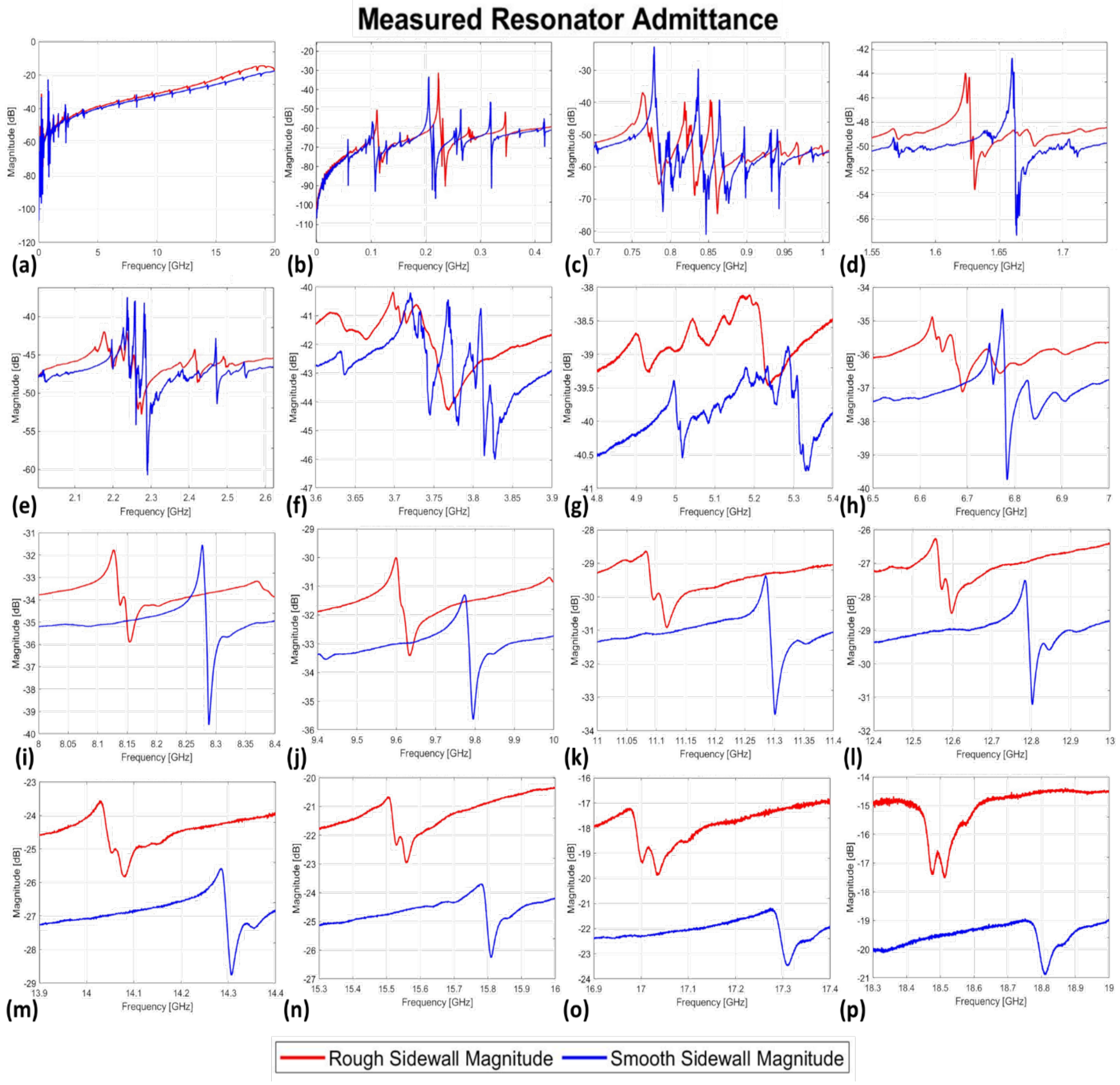

Supplementary Figure S5: (a) Broad frequency sweep of resonator admittance. (b) – (p) Admittance of individual resonance modes.

The scattering parameters (s-parameters) were measured from Keysight network analyzer for the 2 port devices. The admittance measurements in dB scale were calculated from s-parameters using Matlab RF Toolbox.

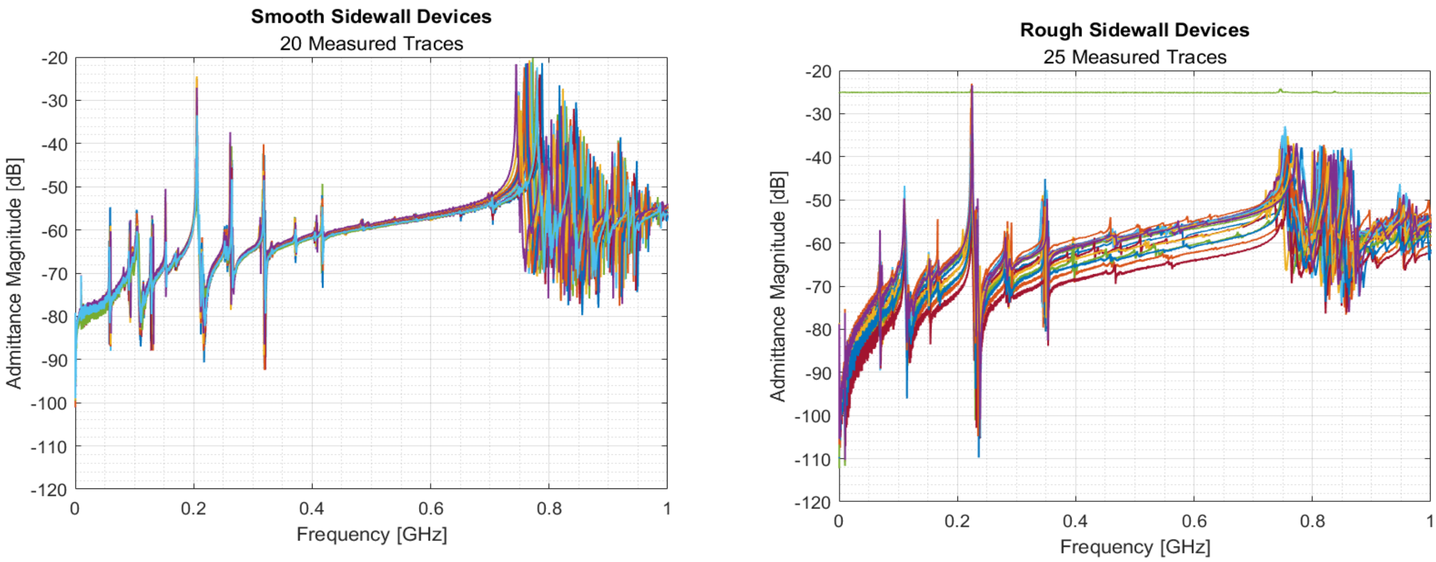

Supplementary Figure S6. All measured frequency responses of the smooth sidewall (left) and rough sidewall (right) designs overlaid on top of each other.

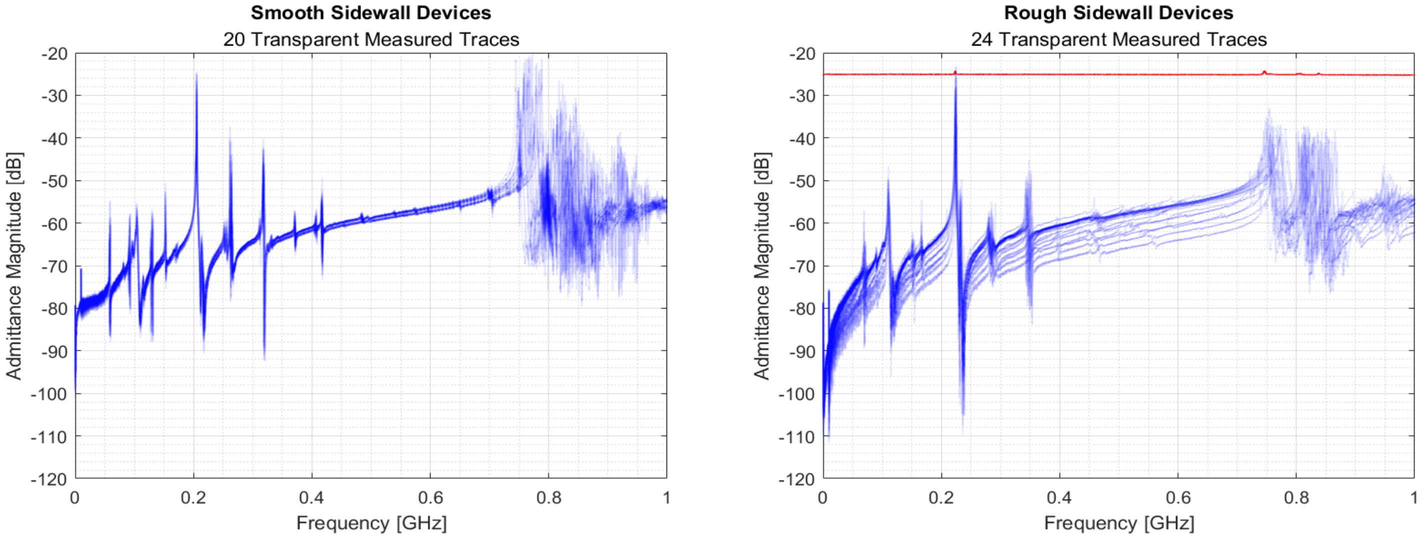

Supplementary Figure S7. Transparent traces of the frequency responses of the smooth sidewall (left) and rough sidewall (right) designs overlaid on top of each other. Darker regions indicate multiple overlapping traces, highlighting similarity between the different devices of the same design. The red traces are outlier devices that did not yield, that are neglected for statistical analysis.

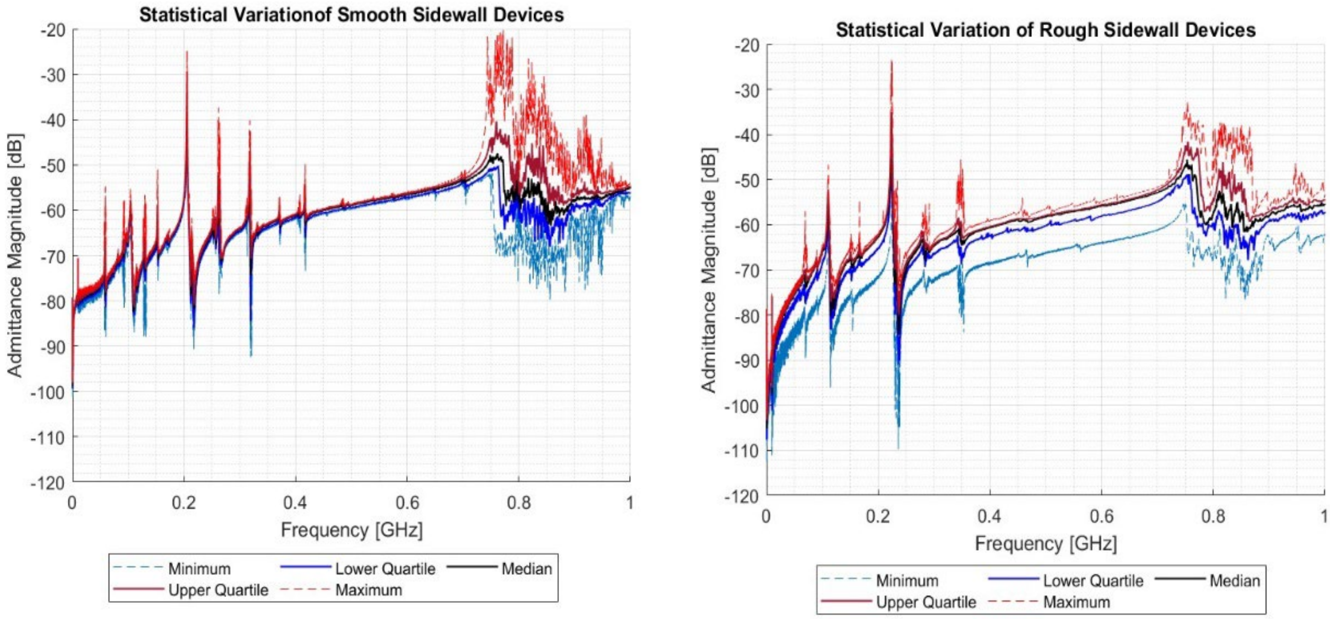

Supplementary Figure S8. Statistical variation of the frequency responses of the smooth sidewall (left) and rough sidewall (right) designs.

COMSOL Multiphysics FEM simulations for the resonator

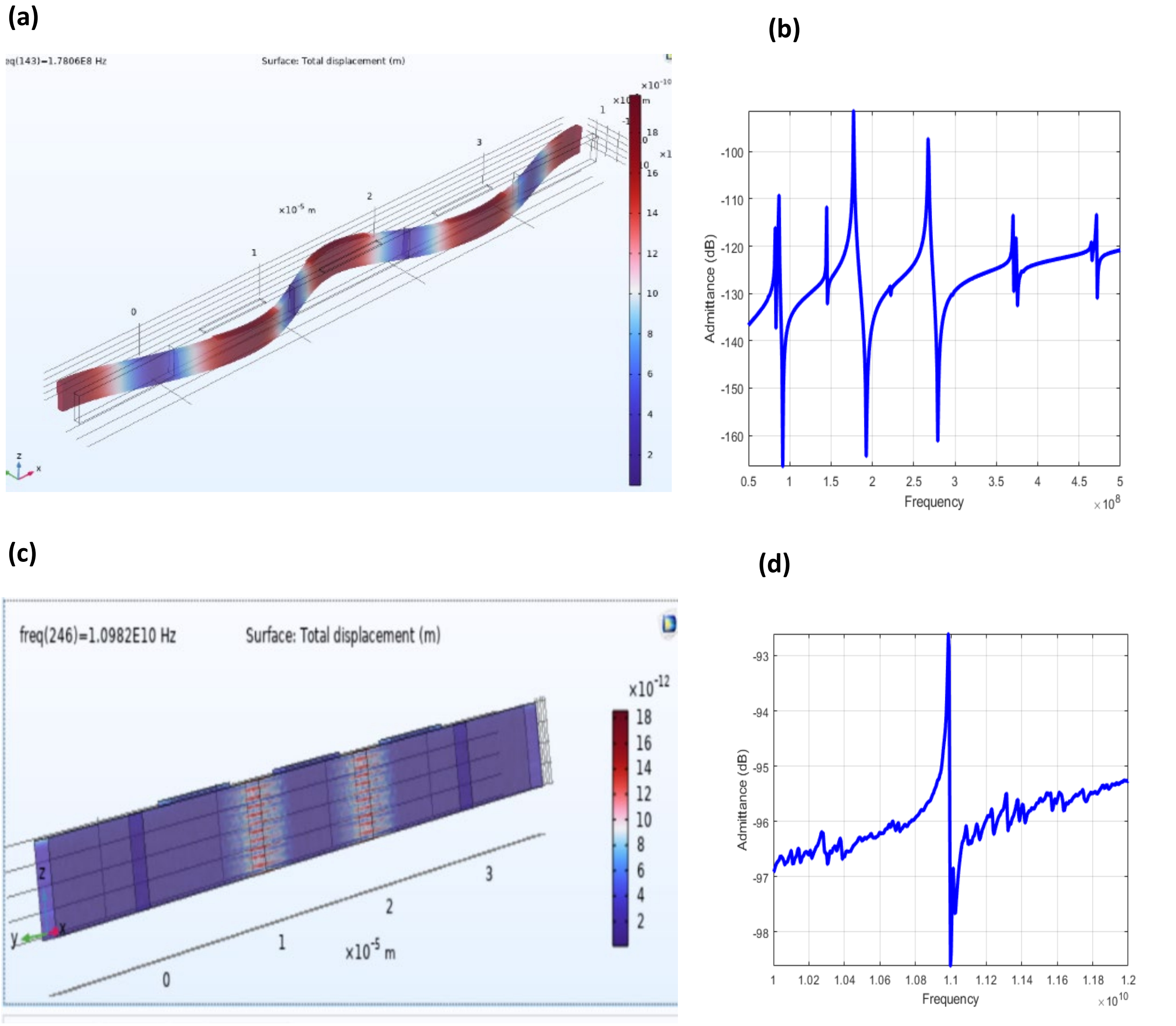

Supplementary Figure S9: (a) Device showing shear horizontal mode at low frequency. (b) Admittance of individual resonance modes at low frequencies. (c) Device showing transverse mode at high frequencies. (d) Admittance of individual resonance modes at high frequencies.

References:

1. Chu, P. B. *et al.* Controlled pulse-etching with xenon difluoride. in *Proceedings of international solid state sensors and actuators conference (Transducers' 97)* vol. 1 665–668 (IEEE, 1997).
